# Fecal metabarcoding vs traditional invasive survey to access the diversity of anuran endoparasites and diet

**DOI:** 10.1017/S0031182025100292

**Published:** 2025-08

**Authors:** Amanda Dudczak, Rafael Euclydes, Emanuel Razzolini, Hernani Fernandes Magalhães de Oliveira, Melissa Ingala, Karla Magalhães Campião

**Affiliations:** 1 Biological Interactions Laboratory, Federal University of Paraná, Curitiba, Brazil; 2 Department of Biological Sciences, Fairleigh Dickinson University, Madison, NY, USA; 3 National Museum of Natural History, Smithsonian Institution, Washington, DC, USA

**Keywords:** amphibia, eDNA, helminth, non-lethal

## Abstract

Identifying interactions between species is essential for understanding ecosystem dynamics. With their central position in trophic networks, anurans underscore the importance of studying their interactions with other organisms. Traditionally, collecting and describing anuran helminth parasites rely on lethal methods, posing challenges for studying threatened species. In this study, we tested the effectiveness of non-invasive fecal metabarcoding and compared its accuracy to traditional invasive methods for identifying parasites and dietary components. We collected anurans from 6 families in the Brazilian Atlantic Rainforest and analysed their feces using the 18S marker while performing necropsies for traditional identification. Traditional methods identified 12 parasite taxa and 3 dietary items at lower taxonomic resolution. Fecal metabarcoding, on the other hand, revealed greater diversity and fine taxonomic resolution for dietary items, although with lower accuracy for parasites due to database limitations. The metabarcoding approach demonstrated a high potential for non-lethal biodiversity assessments, offering a more comprehensive view of dietary diversity and a viable alternative for studying parasites in vulnerable populations. However, its effectiveness depends on improving reference databases, especially for parasite taxa. The advancement of non-invasive approaches that integrate parasitological data holds great potential to improve conservation strategies and enhance the ecological understanding of amphibian-parasite interactions.

## Graphical abstract

**Figure:**
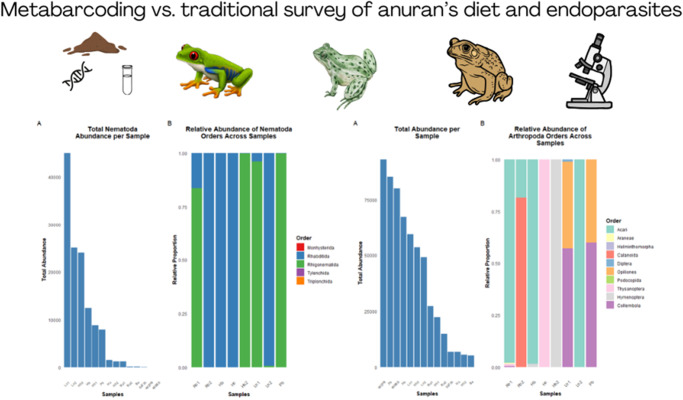

## Introduction

Despite many species extinctions in the last decades, tropical regions still host incredible species diversity (Brown, 2014), with a high number of cryptic species and intricate species interactions (Nadler and Ponce De León, 2011; Condon et al., 2014). Describing the biodiversity of interactions is not a trivial task, as it generally depends on different types of resources, including methodological approaches, financial resources, and specialists’ expertise. For certain groups, such as endoparasites, the task becomes even more complex. These organisms are often overlooked due to their inconspicuous nature, as many reside within other organisms, rendering them difficult to detect and study. Yet, these organisms are recognized as ‘small players with crucial roles’ (Marcogliese, 2004), influencing evolutionary trajectories and ecosystem services (Wood and Johnson, 2015). Therefore, incorporating parasites and their interactions with host species into ecological studies is essential for a comprehensive understanding of how natural systems function (Brooks et al., 2014; Speer et al., 2022).

Parasites are good indicators of healthy environments, and information on their diversity enables the identification of environmental changes (Koprivnikar et al., 2012; Wylezich et al., 2019). Additionally, parasites are important in causing diseases and controlling host populations (Wood and Johnson, 2015). Emergent diseases caused by introduced pathogens and parasites threaten biodiversity, especially in endangered species (Wake and Vredenburg, 2008; Waddle et al., 2024). Therefore, monitoring parasites is also an important proactive measure to detect early signs of disease outbreaks, understand host-parasite dynamics and mitigate potential impacts on biodiversity (Brooks et al., 2014). Amphibians are particularly valuable models for studying species interactions due to their dual life cycle, which links aquatic and terrestrial ecosystems, and their central positions in food webs as predators and prey of various metazoan groups (Dudczak *et al*., 2022). Despite their ecological importance, neotropical amphibians remain underrepresented in global studies, particularly in host–parasite interactions (Herczeg et al., 2021; Martins et al., 2021).

Traditional methods of studying endoparasites often rely on invasive or lethal sampling techniques (Aivelo and Medlar, 2018). However, these approaches are not feasible for threatened hosts, such as many amphibian species, for which population stability is a concern (Gardner, 2001; Anunciação et al., 2024). Consequently, there is a pressing need to develop accurate, non-invasive methods for endoparasite assessment (Budischak et al., 2015). Advances in molecular tools have increased our knowledge of parasite species detection, showing an important complementary power concerning the information produced using traditional methods (Clare, 2014; Wirta et al., 2014; Scholz, 2024).

DNA metabarcoding uses high-throughput sequencing of environmental samples (eDNA) to identify species by amplifying standardized DNA regions with universal primers (Semenov, 2021). The resulting sequences are then compared against publicly available databases for taxonomic identification (Deiner et al., 2017; Johnson 2024). In this context, fecal DNA metabarcoding has emerged as a widely accepted non-invasive method for the accurate and efficient analysis of animal diet (Ando et al., 2020) and parasite diversity (Davey et al., 2023; Miller et al., 2024). This method has been successfully applied to investigate endoparasites of several vertebrate taxa (Miller et al., 2024), but studies on amphibians are scarce (Aivelo et al., 2018). Additionally, considering the importance of host feeding habits in acquiring parasites (Poulin, 2004; Gómez and Nichols, 2013), assessing anurans’ dietary information may be a complementary tool for understanding their ecological interactions that may result in parasitism. In this study, we compare fecal metabarcoding with traditional invasive techniques to assess parasite and dietary diversity in anuran species from the Atlantic Forest. Our objectives include evaluating the strengths and limitations of each method, highlighting their ecological implications and contributing to the growing body of knowledge on amphibian ecological interactions.

## Materials and methods

### Study area and field collections

We collected anurans in 3 different conserved areas within the Atlantic Rainforest in the Paraná state, south of Brazil: Mananciais da Serra (25°29′29″S, 48°59′39″W), Parque Estadual do Pico Marumbi (25°26′42″S, 48°54′58″W) and Parque Estadual Floresta do Palmito (25°35′37″S, 48°33′39″W) in December 2021. Anurans were sampled at night using active visual and auditory searches. After capture, each anuran sample was placed in an individual thermic container at room temperature overnight. The next morning, we searched for and collected feces using sterile gloves that were changed between samples to avoid cross-contamination. The feces were stored in a 1.5 mL Eppendorf tube containing 100% ethanol and kept at −20 °C prior to DNA extraction. The anurans that did not defecate within 24 h were returned to the wild. After collecting feces, we euthanized the anurans using an overdose of lidocaine 4%, following the current Brazilian legislation (Federal Council of Biology – CFBIO, Resolution 308).

### Diet analysis from stomach content survey

We mainly collected stomach contents, and when viable, we also collected diet items from the intestines. These items were kept in 70% ethanol and identified under a stereoscopic microscope up to the order or family level whenever possible. Morphological identification was used to compare our identification with the metabarcoding data of each individual.

### Traditional parasite survey

We necropsied the anurans for helminth parasites and diet items in the intestinal tract and additionally examined the lungs since lungworms release their eggs through the feces and are common parasites in anurans (Campião et al., 2014). All parasites were stored in 70% ethanol until further analysis. To identify the Nematoda individuals, we cleared them with Aman’s lactophenol on non-permanent slides and observed them under an optical microscope. Acanthocephalans were clarified using lactic acid and observed under a light microscope. Parasite identification and nomenclature followed Anderson et al. (2009) and Hodda (2022) for the phylum Nematoda and Amin (1985) for Acanthocephala.

### DNA extraction from fecal samples

We randomly separated approximately 150 mm^3^ of the fecal pellet using sterilized forceps, transferred it to a new 1.5 mL Eppendorf tube and froze it at −80 °C. Using a pestle, we macerated the frozen feces manually for 1–5 min and repeated the process until the feces were completely homogenized. Next, we mixed the macerated feces with 200 μL cetyltrimethylammonium bromide buffer and incubated it for 20 min at 65 °C. Then, we added 500 μL of chloroform, isoamyl alcohol (CIA), and centrifuged at 10°000 rpm for 10 min. The supernatant was removed, and the process was repeated and precipitated overnight. To remove the CIA reagent, we centrifuged the tubes for 10 min at 10,000 rpm, discarded the supernatant, washed the DNA with 500 μL of 70% cold ethanol and centrifuged again. The supernatant was discarded and air-dried, and the DNA was resuspended in ultrapure water.

### Amplification of the 18S rRNA

Amplification of the 18S rRNA gene targeted the V8 and V9 regions. The primers used were SSU_V8F (5′-ATAACAGGTCTGTGATGCCCT-3′) and SSU_1510R (5′-CCTTCYGCAGGTTCACCTAC-3′), chosen for their specificity and efficiency in detecting metazoans, including Nematoda (Bradley et al., 2016). PCR reactions included 1× reaction buffer with MgCl_2_, 0.2 mM of each dNTP (Thermo Fisher Scientific), 0.4 µM of each primer (Integrated DNA Technologies), 1.25 U of high-fidelity Taq DNA polymerase (Phusion, Thermo Fisher Scientific), 50 ng of extracted genomic DNA and ultrapure molecular-grade water (Sigma-Aldrich).

The thermocycling conditions were optimized to maximize specificity and yield as follows: initial denaturation at 98 °C for 3 min, followed by 35 cycles of denaturation at 98 °C for 10 s, annealing at 55–58 °C for 30 s and extension at 72 °C for 30 s, with a final extension at 72 °C for 7 min. Amplicons were purified using the AMPure XP Beads Kit (Beckman Coulter) to remove primers, free nucleotides and non-specific DNA fragments.

### Library preparation and sequencing

Sequencing libraries were prepared using the Nextera XT DNA Library Prep Kit (Illumina), which included amplicon indexing. The libraries were quantified using a Qubit dsDNA HS Assay Kit (Thermo Fisher) and evaluated on a Bioanalyser 2100 (Agilent Technologies) for size and integrity. Sequencing was conducted on an Illumina MiSeq platform, generating paired-end reads (2 × 150 bp) and ensuring robust coverage for taxonomic analysis.

### Bioinformatics and taxonomic classification

Raw sequence data were demultiplexed, quality-filtered using bcl2fastq (Illumina) and imported into QIIME2 2024.10 (Bolyen et al., 2019). The Cutadapt plugin (Martin, 2011) removes adapter and primer sequences. Low-quality reads (*Q* < 20) were discarded, and amplicon sequence variants (ASVs) were inferred using DADA2 (Callahan et al., 2016), which also corrected sequencing errors and removed chimeras. For chimera removal, the parameter p-min-fold-parent over-abundance was set to 3.

Taxonomic classification of ASVs was performed using the Naive Bayes classifier implemented in QIIME2’s ‘feature-classifier classify-sklearn’ module (Bokulich et al., 2018) and trained using the version 138.2 of SILVA database (Yilmaz et al., 2014). Sequences of Nematoda were categorized into ‘parasites’, and Arthropoda as ‘diet’, based on whether the taxa were known as anuran parasites/prey. To enhance the taxonomic resolution for parasite taxa, sequences identified as Nematoda, Rotifera and Platyhelminthes were aligned with the NCBI nt database using BLAST (Srivathsan et al., 2015). The fecal metabarcoding data were cross-validated using traditional morphological identifications.

## Results

We collected anuran individuals of different species, families and localities (Table 1), gathering paired diet data from the stomach contents, endoparasites and feces for metabarcoding analysis. Through traditional morphological survey methods, we identified 3 insect taxa from anuran gastrointestinal diet contents and 12 taxa of metazoan endoparasites. Fecal metabarcoding detected a total of 576.443 quality sequences from all fecal samples, recovering bacteria, protozoa, fungi, plants and metazoan sequence taxa. The percentage of non-chimeric sequences was an average 35.5%, varying from 9.9% to 65.4% (Supplementary Table S1).

**Table 1.** Species classification and localities of the collected anurans in protected areas of the Brazilian Atlantic Rainforest

Anuran family	Anuran species	Locality
Bufonidae	*Rhinella ornata*	Mananciais da Serra
Craugastoridae	*Haddadus binotatus*	PEP – Parque Estadual Floresta do Palmito
Hylidae	*Boana semiguttata*	Mananciais da Serra
	*Boana semilineata*	PEM – Parque Estadual do Pico Marumbi
	*Scinax tymbamirim*	PEP – Parque Estadual Floresta do Palmito
Hylodidae	*Hylodes cardosoi*	PEM – Parque Estadual do Pico Marumbi
	*Hylodes heyeri*	PEM – Parque Estadual do Pico Marumbi
Leptodactylidae	*Leptodactylus notoaktites*	Mananciais da Serra
Odontophrynidae	*Proceratophrys boiei*	Mananciais da Serra

### Diet analysis from stomach content survey and traditional parasitological survey

Diet items were recovered from the stomachs of 7 anurans. The diet comprised arthropods, morphologically identified as Hymenoptera (Formicidae), Coleoptera and Lepidoptera insects.

Using the traditional method, we morphologically recovered 10 nematode morphospecies and identified them up to species (5), genus (5) or family (2), and one Acanthocephala taxon was identified to genus (Table 2). Among the specimens identified generically, we observed 3 nematodes (*Oswaldocruzia* sp., *Rhabdias* sp.1 and *Rhabdias* sp.2) and 1 acanthocephalan (*Anuracanthorhynchus* sp.), which are potentially new species because their morphology did not match that of the described species. Moreover, morphological identification relies on specific characteristics that may not be observed in all specimens. For instance, the 6 specimens of *Oxyascaris* sp. that we found were all females, and thus, specific identification was not possible because it is based on male sexual morphological characters. Similarly, different genera of Cosmocercidae (such as *Aplectana* and *Cosmocerca*) are mainly distinguished by their reproductive organs and cuticular ornamentation surrounding papillae in the posterior part of males. Therefore, when only females were present in the sample, identification beyond family was not possible. Specimens of Physalopteridae were found in larval stages and therefore lacked adult morphological traits that allow better taxonomic resolution. One host, *Scinax tymbamirim*, did not have any parasite found in the morphological search.

**Table 2.** Morphological survey of anuran parasites in the Brazilian Atlantic rainforest. For each anuran host species, we provide the proportion of infected hosts (NC/NI) and the Mean Intensity of Infection (MII) for each parasite species

Anuran host species	Parasite identification	NC/NI	MII (range)
*Rhinella ornata*	*Aplectana macintoshii*	01/02	9
	*Cosmocerca brasiliensis*	01/02	5
	Cosmocercidae gen. sp.	01/02	7
	*Oswaldocruzia* sp.	02/02	31.5 (9−54)
	Physalopteridae gen. sp.	01/02	1
*Haddadus binotatus*	Cosmocercidae gen. sp.	01/01	4
	*Oswaldocruzia* sp.	01/01	1
*Boana semiguttata*	Cosmocercidae gen. sp.	01/01	1
	*Oxyascaris* sp.	01/01	1
*Boana semilineata*	*Oswaldocruzia* sp.	01/01	1
*Hylodes cardosoi*	*Rhabdias* sp.1	01/01	1
*Hylodes heyeri*	Cosmocercidae gen. sp.	03/03	6.6 (2−12)
	Physalopteridae	01/03	12
	*Rhabdias* sp.2	02/03	7.5 (2−13)
	*Anuracanthorhynchus* sp.	03/03	3.3 (1−4)
*Leptodactylus notoaktites*	*Oxyascaris* sp.	01/02	5
	*Rhabdias* af. *elegans*	01/02	4
	*Schrankiana formosula*	02/02	263 (26−500)
*Proceratophrys boiei*	*Cosmocerca brasiliensis*	02/02	4 (2−6)
	*Oswaldocruzia* sp.	01/02	25
	*Rhabdias megacephala*	01/02	3

NC/NI = number of collected/number of infected; MII, mean intensity of infection with range.

### Diet analysis from feces metabarcoding

**Figure 1.**
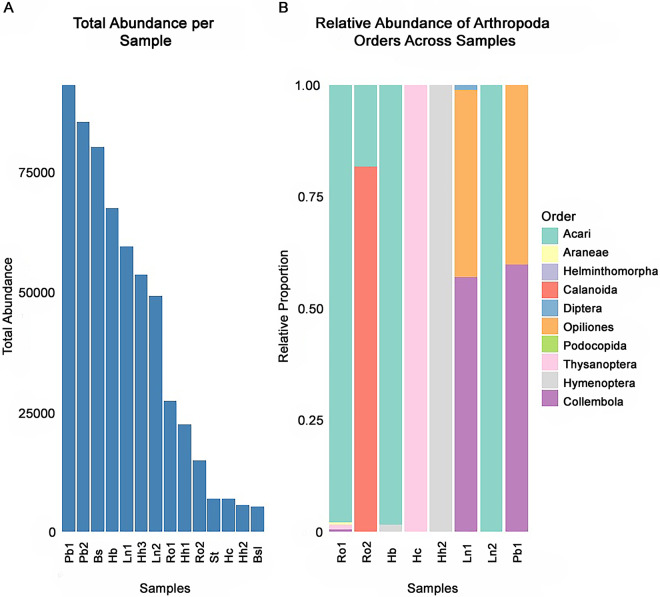
Fecal metabarcoding of anurans’ diet. (A) Total quality sequence abundance of arthropods; (B) relative abundance of arthropods across samples. Each bar corresponds to an anuran specimen, *Boana semiguttata*: Bs; *Boana semilineata*: Bsl; *Haddadus binotatus*: Hb; *Hylodes cardosoi*: Hc; *Hylodes heyeri*: Hh1, Hh2, Hh3; *Leptodactylus notoaktites*: Ln1, Ln2; *Proceratophrys boiei*: Pb1, Pb2; *Rhinella ornata*: Ro1, Ro2; St: *Scinax tymbamirim.*

The feces of 10 anurans presented sequences of Arthropoda that could be assigned as diet, which represent 10.9% of all quality sequences, and were taxonomically classified as Insecta, Arachnida and Maxillopoda (Figure 1). Molecular sequencing of feces revealed the presence of a greater diversity of arthropods ingested by anurans than we observed in their stomachs. The taxonomic assignment provided by QIIME2 showed higher accuracy than the traditional survey and morphological identification. A larger proportion of diet sequences referred to the Arachnida class (42%), with the majority being Acari (36%), followed by Hexapoda (22%) (Figure 1). Most of the taxonomic identification of the sequences reached at least the subclass level, while other substantial proportions reached order (31.31%).

### Parasite survey from feces metabarcoding

**Figure 2.**
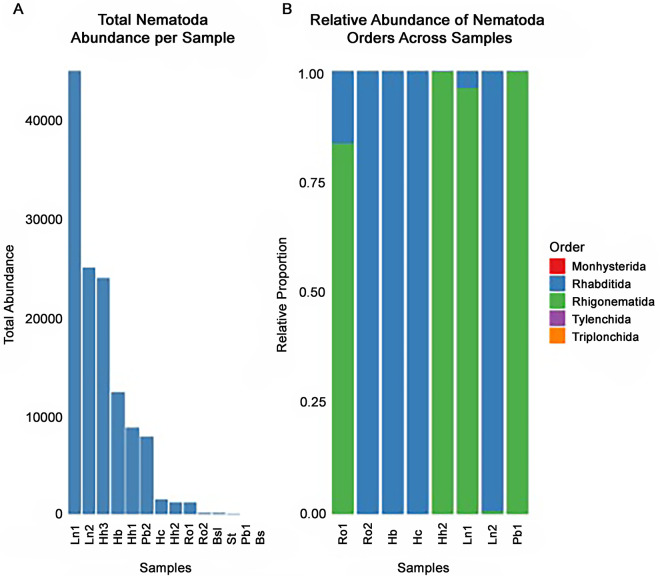
Fecal metabarcoding of anurans’ parasites. (A) Total quality sequence abundance; (B) relative abundance of nematodes across samples. Each bar corresponds to an anuran specimen, *Boana semiguttata*: Bs; *Boana semilineata*: Bsl; *Haddadus binotatus*: Hb; *Hylodes cardosoi*: Hc; *Hylodes heyeri*: Hh1, Hh2, Hh3; *Leptodactylus notoaktites*: Ln1, Ln2; *Proceratophrys boiei*: Pb1, Pb2; *Rhinella ornata*: Ro1, Ro2; St: *Scinax tymbamirim.*

Molecular analysis showed the presence of Nematoda for all, except 2 anuran specimens (Figure 2), including a specimen for which we did not detect any macroparasite in the morphological search. Moreover, most parasite taxa identified morphologically in the intestines of anurans were detected through fecal metabarcoding but were mostly identified up to the order level (Figure 2; Supplementary Table S2). The taxonomic classification of the Nematoda phylum recovered sequences from the classes Chromadorea (orders: Rhabditida and Monhysterida) and Enoplea (orders: Enoplida and Triplonchida). Only one nematode taxon was identified at the species level: the free-living nematode *Prismatolaimus intermedius* (Enoplea: Triplonchida), which was not observed in the morphological search. Fecal metabarcoding also recovered sequences from Platyhelminthes and Rotifera, but they matched free-living instead of parasite taxa.

For the second step of sequence identification, we used BLAST searches of the metabarcode sequences of the Nematoda, Platyhelminthes and Rotifera taxa against the GenBank database and recovered a more specific classification. While the platform QIIME2 only identified parasites to the order level (Rhabditida), GenBank could match those sequences with *Strongyloides* sp., *Oswaldocruzia* sp. and *Rhabdias* sp. (Query Cover: >99% and per. ident: >97.8), and specimens of the last 2 genera were indeed present in the anuran intestines and lungs, respectively, according to our morphological search. Similarly, BLAST searches of the sequences identified as belonging to the class Chromadorea by QIIME2 against GenBank matched them with Cosmocercidae, *Cosmocerca* sp. and *Aplectana* sp. (Query Cover: 100%; per. ident: >99.2), which were also confirmed by our results of the morphological search (Table 2). We observed species of the genera *Oxyascaris* and *Schrankiana*, but neither QIIME2 nor GenBank BLAST searches matched the sequences of these taxa.

### Comparing morphological vs metabarcoding for metazoan parasite detection and identification

Among the metazoans, we detected Nematoda, which were identified mostly up to the order level. In the first step of sequence identification, which is more comprehensive, we observed that only a part of the nematode sequences was composed of parasite taxa, while the others were identified as free-living, as well as Platyhelminthes and Rotifera (Supplementary Table S2). In the second step of sequence identification, we selected sequences assigned to Nematoda, Platyhelminthes and Rotifera and compared them against the GenBank database. The Nematoda sequences matched several parasite taxa, some unrelated to the surveyed hosts, while others included genera that we had also identified morphologically to the genus level.

A small number of sequences were identified as Platyhelminthes in the metabarcoding analysis (Supplementary Table S2), and we did not find any parasite taxa of Platyhelminthes in the intestines. Moreover, we found acanthocephalans parasitizing the individuals of *Hylodes heyeri* through morphological analysis, while the metabarcoding analysis recovered sequences corresponding to free-living rotifers, specifically Bdelloidea and Monogononta. Interestingly, the rotifer sequences were found exclusively in the same anurans where acanthocephalans were detected morphologically. This match suggests that the rotifer sequences recovered in the metabarcoding analysis could represent misidentified acanthocephalans, likely due to phylogenetic proximity (García-Varela and Nadler, 2006) and the sequencing of conserved genetic regions shared between these taxa.

## Discussion

This study is the first to compare parasite and diet diversity in anurans using DNA fecal metabarcoding paired with traditional morphological methods. Metabarcoding revealed a greater diversity of organisms than was observed through morphological analysis. Fecal metabarcoding captured a higher diversity of arthropods that are likely dietary items, being more efficient than traditional invasive methods in detecting a wide spectrum of dietary diversity in anurans. However, it did not entirely align with stomach content findings. The method also proved effective for parasite identification; however, it still relies on expert input, as the lack of a robust genetic sequence database can lead to spurious matches (Clarke et al., 2014). For example, in a host-parasitoid system, only 39.4% of parasitoid sequences could be identified to the species level, compared with 90% of invertebrate hosts, suggesting that parasite diversity may be missed or underestimated depending on how much genetic sampling has been done on that group (Šigut et al., 2017).

Fecal metabarcoding identified taxa such as Nematoda, Platyhelminthes and Rotifera, often to the order level, while several arthropods were identified to the species level. The superior taxonomic resolution for dietary items compared to parasites reflects the extensive representation of arthropod sequences in GenBank. However, as discussed earlier, the limited availability of reference sequences for parasite taxa in GenBank remains a challenge. This contrast highlights the need for targeted efforts to expand molecular databases for underrepresented groups like Nematoda, Platyhelminthes and Acanthocephala (Scholz, 2024). Expanding reference databases for parasite taxa, especially in Neotropical regions, is critical to enhancing metabarcoding utility (Huggins et al., 2017; Budischak et al., 2018).

A trade-off exists between accuracy and detectability in genetic analysis (Srivathsan et al., 2015; Aivelo and Medlar, 2018). Specific molecular markers enhance taxonomic accuracy but limit the range of taxa detected, whereas universal markers like the 18S rRNA maximize detectability across diverse groups (Blasco-Costa et al., 2016). In this study, the use of a broad-spectrum marker (Peham et al., 2017) was crucial for detecting both known and potentially undescribed parasites, given the limited molecular data available for most anuran parasites (Huggins et al., 2017; Budischak et al., 2018). This approach ensures that even when the target taxon is absent in databases, closely related species can be identified (Mande et al., 2012). However, highly conserved sequences may limit taxonomic resolution (Dueholm et al., 2020), as demonstrated by BLAST analyses of Rhabditida sequences, which matched multiple genera and required careful interpretation to exclude irrelevant taxa. Metabarcoding provides an efficient overview of parasite diversity, as observed in studies on other taxa, such as wild rats (Tanaka et al., 2014; Hino et al., 2016), wolverines (Watson et al., 2020) and primates (Aivelo and Medlar, 2018). However, its effectiveness depends on continued efforts to enhance reference libraries and develop targeted molecular markers (Blasco-Costa et al., 2016). Moreover, using group-specific primers in follow-up analyses can refine taxonomic resolution for targeted groups, such as nematodes, and catch potentially spurious matches from genetics alone (Alberdi et al., 2018).

Arachnids were the most common dietary items in the fecal metabarcoding but were not observed in the morphological survey of stomach contents; however, the interpretation of abundance estimates from fecal metabarcoding may be taken cautiously (Stapleton et al., 2022). Moreover, mismatches between fecal metabarcoding and stomach contents may probably reflect temporal turnover, reflecting differences between recent predation events and longer-term dietary history. Anurans’ opportunistic predation habits likely contribute to these differences (Rodrigues and Santos-Costa, 2014). In addition, one limitation of cross-sectional diet metabarcoding is that it only represents a ‘snapshot in time’ of what an animal eats, making such studies limited in scope. However, repeated sampling throughout seasons and years, or combination with longer-term diet assessment methods such as stable isotopes, can give a longitudinal view of diet and begin to approximate the overall dietary niche of the species (e.g., Soininen et al., 2014; Oelbaum et al., 2019). Indeed, fecal metabarcoding has been increasingly and successfully used to describe vertebrate diets, with the identification of even rare items (Stapleton et al., 2022; Yoshimura et al., 2024).

Anurans are preyed upon by a wide range of vertebrates and host a diverse community of trophically transmitted parasites (Koprivnikar et al., 2012). These parasites are often encysted and, like those infecting non-intestinal organs (e.g., blood parasites such as microfilariae), are not detectable via fecal metabarcoding. Moreover, males and immature individuals of parasites are not detected by this method, and for females, the amount of DNA detected may be influenced by factors such as egg load. Therefore, quantitative assessment of parasite infection intensity is limited, as metabarcoding estimates of parasite abundance may not accurately reflect reality (Stapleton et al., 2022).

The small sample size is a methodological limitation of our study, although the consistent patterns observed across individuals and taxa underscore the robustness of our findings and their relevance to broader applications. The sample size also aligns with the ethical constraints, as our methodological approach included the evaluation of invasive methods on endemic species in protected areas. Additionally, fecal metabarcoding is susceptible to environmental contamination and can also account for accidental intake by the organisms studied. This probably happened in our study, since anurans are sloppy feeders and accidental ingestions may occur. Future studies should incorporate template controls, which can be filtered bioinformatically to better detect primer bias (Ficetola et al., 2016; Van der Loos and Nijland, 2021; Sickel et al., 2023).

Our results demonstrated the complementary nature of fecal metabarcoding and traditional endoparasite survey methods for metazoan parasite identification. Morphological analysis is essential for identifying novel or poorly represented taxa, such as the putative new species found in this study. Metabarcoding, in turn, is valuable for detecting a broad range of taxa – from bacteria and protozoans to metazoans – and is particularly useful for non-invasive assessments of rare or endangered host species. This combination of approaches can significantly improve biodiversity assessments. Considering both the advantages and disadvantages mentioned here, we believe fecal metabarcoding is suitable for exploratory studies aimed at surveying parasite and dietary diversity across host populations, especially in cases where traditional methods are infeasible due to ethical or logistical constraints.

The primary aim of this study was to evaluate the accuracy of detecting and identifying interactions between invertebrates and anurans using fecal metabarcoding compared to traditional morphological surveys. We found differences in the identification accuracy between the these approaches for both anuran parasites and diet. While initial taxonomic identification through metabarcoding typically resolved sequences to the order level, the use of database tools can provide more specific identifications. This study represents an important first step in the development of a non-invasive method for identifying parasitized anurans. By enabling the detection of parasite–host interactions without the need for lethal sampling, this approach opens new avenues for future research. It holds potential for monitoring infected hosts and exploring correlations between parasitism, host behaviour, reproduction, survival and conservation threats, particularly in vulnerable populations.

## Supporting information

Dudczak et al. supplementary material 1 Dudczak et al. supplementary material

Dudczak et al. supplementary material 2 Dudczak et al. supplementary material

## Supplementary material

The supplementary material for this article can be found at https://doi.org/10.1017/S0031182025100292.
